# CD28 ^null^ CD4 T-cell expansions in autoimmune disease suggest a link with cytomegalovirus infection

**DOI:** 10.12688/f1000research.17119.1

**Published:** 2019-03-25

**Authors:** Aalia Bano, Alejandra Pera, Ahmad Almoukayed, Thomas H.S. Clarke, Sukaina Kirmani, Kevin A. Davies, Florian Kern

**Affiliations:** 1 Department of Clinical and Experimental medicine, Brighton and Sussex Medical School, Brighton, Sussex, BN1 9PX, UK; 2 Department of Immunology, Maimonides Institute for Biomedical Research (IMIBIC), Reina Sofia Hospital, University of Cordoba, Av. Menendez Pidal, 14004, Cordoba, Spain

**Keywords:** CD28 null, ​​​​​​​CD4 T cell, autoimmune disease, cytomegalovirus infection

## Abstract

Immunosenescence is thought to contribute to the increase of autoimmune diseases in older people. Immunosenescence is often associated with the presence of an expanded population of CD4 T cells lacking expression of CD28 (CD28 ^null^). These highly cytotoxic CD4 T cells were isolated from disease-affected tissues in patients with rheumatoid arthritis, systemic lupus erythematosus, multiple sclerosis, or other chronic inflammatory diseases and their numbers appeared to be linked to disease severity. However, we recently demonstrated that the common herpes virus, cytomegalovirus (CMV), not ageing, is the major driver of this subset of cytotoxic T cells. In this review, we discuss how CMV might potentiate and exacerbate autoimmune disease through the expansion of CD28 ^null^ CD4 T cells.

## Autoimmunity is not a rare phenomenon

A degree of autoimmunity that is, reactivity of the immune system against our own tissues (‘self’) is found in everybody. This is because the immune system’s ability to discriminate ‘self’ and ‘non-self’ is not perfect. However, this imperfection usually is not enough to cause autoimmune disease. It appears that autoimmune disease requires “a genetic predisposition and environmental factors that trigger the immune pathways that lead, ultimately, to tissue destruction” ^1^. This review article was prompted by the recent (renewed) interest in CD28 ^null^ CD4 T cell–driven tissue damage and focuses on the adaptive immune system. In the following sections, we explore some of these factors and a possible environmental trigger for some autoimmune diseases. Manifestations of autoimmunity may occur in almost any tissue. In someone who has a predisposition to autoimmune disease and is exposed to the respective trigger, the immune system, however, still has ways of regulating itself in order to prevent tissue damage. Some researchers would argue that autoimmune disease ultimately results from an imbalance between effector and regulatory immune responses (that is, between a damaging response and another one keeping it under control). This means that immune regulation may still prevent the occurrence of autoimmune disease even if an immune response against self is present. Disease occurs only if the mechanisms preventing tissue damage are no longer sufficient. This may happen, for example, if the regulatory response for some reason ceases to work or the damaging response is boosted.

## Predisposition to autoimmune disease

There are many different factors facilitating the development of autoimmune disease. Among genetic defects, primary immunodeficiencies are often associated with autoimmunity and clinical manifestations thereof. Complement deficiencies, for example, have long been known to be risk factors for lupus ^2^. While infections often are considered the main consequences of primary immunodeficiencies, such defects may also interfere with immune regulation (for example, in preventing autoreactive T cells and B cells from being reined in by regulatory cells). An interesting example is a life-threatening autoimmune condition known as ‘IPEX syndrome’ (immune dysregulation, polyendocrinopathy, enteropathy, X-linked syndrome). It is caused by mutations affecting the ‘master regulator’, forkhead box P3 (FOXP3) gene, an important transcription factor in regulatory T cells (Tregs) ^3^. As a result, there is loss of CD4 T-cell regulation, resulting in the production of autoantibodies and tissue destruction (the role of Tregs in peripheral tolerance is discussed below in the ‘Tolerance versus autoimmunity’ section). An example for an acquired predisposition to autoimmune diseases is HIV infection. The incidence of rheumatic autoimmune diseases observed in HIV infection (which is an acquired immunodeficiency) was relatively low during the era before highly active anti-retroviral therapy (HAART). However, following the introduction of HAART, a dramatic increase of conditions linked to an increase in CD4 T-cell counts was observed, which underscores the important role of CD4 T cells as effectors in such autoimmune diseases ^4, 5^. A genetic predisposition, importantly, does not have to be a defect since many autoimmune diseases are associated with major histocompatibility complex (MHC) type. CD4 T cells recognise peptide antigens in the context of class II MHC molecules, and the mere presence of certain MHC alleles/allele groups may predispose an individual to autoimmune disease. One explanation for such associations is that MHC molecules allow specific, disease-associated peptides to be presented to CD4 T cells. Such a mechanism, for example, was discovered with respect to several HLA-DQB1 (‘DQ2’ and ‘DQ8’) alleles in gluten-sensitive enteropathy (‘coeliac disease’). The association in this case is so strong that the absence of DQ2 and DQ8 essentially rules out the disease ^6^. However, it is still only a minority of individuals with that tissue type who develop autoimmune disease.

## Tolerance versus autoimmunity

Tolerance is a state where the immune system tolerates the presence of antigens that are recognised by immune cells without mounting an attack on the tissues displaying them. Whereas passive tolerance (essentially a gap in the immune receptor repertoire) is rare and refers to an absence of antigen recognition, active tolerance implies that mechanisms operate that actively down-regulate or suppress an otherwise detrimental mechanism. So-called central tolerance (or recessive tolerance) is achieved by eliminating all T lymphocytes that react with ‘self’ antigens during a rigorous selection process in the thymus. T cells bearing T-cell receptors (TCRs) that are autoreactive in the context of self-MHC molecules are deleted or may be diverted to become Tregs ^7^. However, ‘perfect’ central tolerance would require the presence of all relevant self-antigens in the thymus in sufficient quantity at the time of TCR selection. As this does not seem to be possible, some autoreactive cells tend to slip through the selection process ^8^. As a result, additional, ‘peripheral’ tolerance mechanisms are required to regulate inappropriate activation of autoreactive T cells in the periphery. Several different mechanisms contribute to peripheral tolerance.

The first important safeguard against the activation of autoreactive T cells is the requirement of two signals to induce T-cell activation. The interaction of TCRs with MHC/peptide complexes on antigen-presenting cells (APCs) (signal 1) alone is insufficient to cause T-cell activation. A second, co-stimulatory signal is required for full activation. This is usually provided by surface ligands called CD80 and CD86 on APCs. These interact with the co-stimulatory receptor, CD28, on T cells ^9– 11^. In the absence of the second signal, T cells become hypo-responsive and undergo apoptosis. A second safeguard is provided by the expression of the surface receptor molecule, cytotoxic T lymphocyte–associated protein 4 (CTLA-4), on activated T cells. Its affinity for CD80/CD86 is higher than that of CD28 and interaction between the former and CTLA-4 on effector T cells dampens down their activity. In addition, CTLA-4 may remove CD80/86 from APCs. There is strong evidence in the literature that regulating CD28-dependent T-cell activation is the most important role of CTLA-4 ^12^. Tregs express CTLA-4 constitutively and its expression is further stabilised by FOXP3 expression, a ‘hallmark’ of Tregs. The effect of CTLA-4 ligation on Tregs seems to be an enhancement of suppressor activity. Whether or not the actual signalling through CTLA-4 has any role in this process remains controversial. However, suppressor activity of Tregs may keep the activation of cytotoxic T cells by self-antigens under control ^13, 14^.

In some situations, an environmental trigger can breach existing tolerance. This can occur by ‘unmasking’ epitopes that were not present during selection in the thymus. This is one of the postulated mechanisms by which enterovirus infection might trigger autoimmune diabetes ^15^. In this review, however, we focus on a different virus, cytomegalovirus (CMV), as a possible trigger of autoimmune disease. CMV is a common herpes virus. The precise mechanisms by which it might trigger autoimmunity have remained obscure but its association with autoimmune disease is striking ^16^.

## The nature of CD28 ^null^ T cells

The loss of CD28 associated with cellular senescence is persistent and must not be confused with the short-lived down-regulation of CD28 after T-cell activation, which usually reverses within 48 hours ^17^. Whereas non-antigen-experienced T cells express the co-stimulatory surface receptor, CD28, following the encounter of their cognate antigen, antigen-experienced T cells proliferate and differentiate along a pathway of increasing maturity that eventually leads to the loss of CD28, at least in CD8 T cells. Repeated cell division leads to telomere shortening down to a critical length below which cells are pushed into ‘replicative senescence’ and ultimately programmed cell death (apoptosis). Cells that have lost part or all of their ability to divide are considered senescent. CD28 ^null^ T cells are ‘terminally differentiated’, have shortened telomeres and are often classified as senescent. They no longer require CD28 ligation for complete activation and at the same time have great cytotoxic potential ( Table 1). The loss of CD28 expression on both CD4 and CD8 T cells was long considered to be a consequence of normal (immune system) ageing, providing an explanation for accumulations of CD28 ^null^ T cells in older people ^18^. For example, Effros *et al*. reported that significantly decreased populations of CD28 ^+^ cells in centenarians compared with younger people and a loss of CD28 ^+^ T cells in long-term culture appeared to indicate that loss of CD28 is a direct consequence of immunosenescence ^19^.

**Table 1.** CD28 ^null^ CD4 T-cell associations with different clinical conditions.

Disease	Characteristics of CD4 ^+^28 ^−^ T cells	Invasion of disease-specific tissue	Correlation to disease and therapies	Possible self-antigens (residues) sharing sequences with cytomegalovirus (CMV) peptides	References
Multiple sclerosis (MS)	Pro-inflammatory (interferon-gamma [IFN-γ], tumour necrosis factor-alpha [TNF-α], and interleukin-2 [IL-2]) IL-15 amplifies pathogenic properties in patients Fully potent regulatory T cells can suppress their pro-inflammatory features but not their expansion	Cerebrospinal fluid and brain lesions	Worse outcome in relapsing-remitting MS (RRMS) More frequent evoked potentials in RRMS Worse Expanded Disability Status Scale (EDSS) score	CMV major capsid protein UL86 (981–1003) and MOG (34–56) Myelin basic peptide (93–105) and human herpesvirus-6 U24 (1–13)	20, 29, 41, 42, 43– 47
Rheumatoid arthritis (RA)	Proliferation mediated by fractalkine- expressing synoviocytes Dependent on chronic exposure from TNF- α/IL-15, resulting in limited T-cell receptor (TCR) diversity Pro-inflammatory (IFN-γ and TNF-α) In synovium, expresses chemokine receptors (CX _3_CR1) Activates natural killer cell receptors (CD11b, CD57, and NKG2D)	Synovial fluid of affected joints Lung infiltration of CD4 ^+^ in RA-associated pneumonitis	Preclinical atherosclerotic changes (endothelial dysfunction and carotid artery wall thickening) Disease severity positively correlates with amount of subset present Pre-disposition to extra-articular inflammatory lesions Infliximab reduces CD4 ^+^CD28 ^−^ T cells	Human collagen Type II (associated with HLA- DRB1*0401 and HLA- DQA1*0301-DQB1*0302	32, 48, 49, 50, 51, 52– 58
Graves’ disease	Pro-inflammatory (IFN-γ) Mainly express CD45RO (activated/memory T cells)	Eye muscle tissue, orbital fat tissue, and thyroid tissue	Severity of Graves’ disease Severity of goitre Serum FT3 and TRAb levels positively correlate Increased intracellular IFN-γ secretion in patients with Graves opthalmopathy Increased anti-thyrotropin receptor antibodies		22, 59, 60
Systemic lupus erythematosus (SLE)	Pro-inflammatory (IFN-γ) Increased percentage of CD4 ^+^NKG2D ^+^ T cells Expression is induced by SLE monocytes	Skin lesions	Inversely correlates with regulatory T cells Disease severity (using both Systemic Lupus Erythematosus Disease Activity Index 2000 [SLEDAI-2K] and Systemic Lupus International Collaborating Clinics/American College of Rheumatology Damage Index [SDI])	CMV J1S = VASRPL F P PRSPGPS and HRES1 = PRHRHPQDPRSPGPA CMV protein gB and the autoantigen U1 70k C terminal half of HCMVpp65 and anti-nuclear antibodies (BALB/C mice)	61– 66
Cardiovascular diseases	Expression of killer immunoglobulin-like receptors (KIRs) (that is, KIR2DS2) Destabilising fibrous caps of atherosclerotic plaques Release of perforin and granzyme B causes lysis of endothelial cells and vascular smooth muscle damage (without TCR stimulation)	Preferential invasion of unstable plaques	Severity of atherosclerosis and unstable angina (UA) Acute coronary events in patients with UA Reduced by statin therapy	hHSP60 _153–163_ with CMV UL122 and CMV US28	57, 67– 69

## Immunosenescence, CD28 ^null^ CD4 T cells, and autoimmunity

Large populations of CD28 ^null^ CD4 T cells were first identified in rheumatoid arthritis (RA) and later found in a wide variety of autoimmune diseases ranging from multiple sclerosis (MS) to Graves’ disease (GD) ^20– 22^. However, the loss of CD28 does not seem to indicate ‘anergy’. On the contrary, these cells acquire atypical cytotoxic properties resulting in a more rapid response. CD28 ^null^ CD4 T cells have been described to have a memory effector phenotype and hence do not require a co-stimulatory input to be re-activated ^23^. They are also unusual in having a large cytoplasmic store of granzymes and perforins characteristic of CD8 T cells and natural killer (NK) cells ^20^. In addition, they produce large amounts of pro-inflammatory cytokines such as interferon-gamma (IFN-γ) and tumour necrosis factor (TNF), which potentially contribute to host tissue damage. Finally, CD28 ^null^ CD4 T cells express NK cell markers such as NKG2D, which possibly further contribute to the amplification of the pro-inflammatory signals ^24^. These extra receptors also lower the threshold for the activation of CD28 ^null^ CD4 T cells by specific and non-specific stimuli ^25^.

Interestingly, CD28 ^null^ CD4 T cells are resistant to multiple forms of regulation. They are resistant to CD4 ^+^CD25 ^+^Treg-mediated suppression, a peripheral tolerance mechanism inhibiting proliferation and cytokine production. Tregs were shown to be unable to restrict the proliferation of CD28 ^null^ CD4 T cells because of the lack of IL-2 receptor molecules (CD25) on these cells ^26– 28^. They are also resistant to apoptosis because of the down-regulation of the first apoptosis signal Fas receptor (Apo-1) and up-regulation of the B-cell lymphoma 2 (Bcl-2) apoptosis regulator. Both of these mechanisms contribute further to their accumulation ^29^. When CD28 ^null^ T cells become activated, they rapidly express CTLA-4 and generate a pro-survival signal by reducing caspase-mediated apoptosis. Anti-apoptotic resistance is also rendered by B7.1 and B7.2/CTLA-4 interaction since blocking CTLA-4 increases Fas-FasL-dependent apoptosis ^30^. In light of these properties, expansions of CD28 ^null^ CD4 T cells would be expected to be able to drive regulation-resistant tissue destruction.

## The ‘reversible’ nature of immunosenescence and CD28 loss

According to Vallejo *et al*., persistent loss of CD28 most probably depends on repeated or continuous antigen contact ^17^. Since CD28 loss on T cells is frequently implicated in the development of immunosenescence, it is of interest that others have also observed that chronic, persistent antigen stimulation (as seen in chronic viral infection or chronic inflammatory/autoimmune disorders) accelerates immunological ageing ^31, 32^. CMV and HIV infections are prime examples of chronic virus infections associated with premature immune senescence ^33– 36^ but hepatitis B virus also appears to be in that category ^37^. These observations raise the question as to whether cellular senescence might be reversible if the continuous antigenic stimulation were abrogated. And if so, might autoimmunity associated with CD28 ^null^ CD4 T cells be reversible? Warrington *et al*., back in 2003, showed that CD28 loss on CD28 ^null^ CD4 T cells can be reversed *in vitro* by stimulation with IL-12 ^38^.

More recently, Akbar *et al*. reported that cellular senescence is an active process that is (at least partly) reversible ^39^. They showed that, apart from telomere erosion and DNA damage, a nutrient-sensing pathway is also responsible for activating p38 mitogen-activated protein (MAP) kinase in senescent cells. They also showed that small-molecule inhibitors of p38 reconstitute proliferation and telomerase activity following T-cell activation ^39^. Other studies showed that, in addition to these intrinsic pathways, T-cell senescence can be reversed extrinsically by cytokines secreted by APCs. A recent study in mice showed that p38 MAP kinase inhibition in APCs led to an increase in interleukin-12 (IL-12) production, which in turn enhanced T helper 1 (Th1) responses after vaccination in a mouse model ^40^. It is quite possible that in humans the effect of p38 MAP kinase inhibition on T cells ^39^ is mediated by IL-12 as well.

The concept that cellular senescence is an active and reversible process is very confusing per se, since generally we associate the term ‘senescence’ with chronological age (which certainly is not reversible). Therefore, it is important to understand that senescent cells do not have to be ‘old’ in a chronological sense and may occur in young people. The fact that an increase in cells with an apparently ‘senescent’ phenotype is often associated with older age is explained by the accumulation of antigen-experienced cells in older people. Many of these will have been exposed to their cognate antigens for a long time and have gone through many rounds of proliferation. However, it is important to consider that fully functional, terminally differentiated effector cells would also be expected to produce aggressive cytokines (that is, have a secretory phenotype) without necessarily being senescent. The CD28 ^null^ phenotype might include both senescent and fully functional effector cells. However, independently of these phenotypes, the age of an organism might have an effect on cellular function in general. Recent data suggest that, for example, naïve T cells in older people already show some signs of senescence ^70^.

## CD28 ^null^ CD4 T cells are strongly associated with cytomegalovirus infection

Because the prevalence of CMV infection increases with age, it was widely believed that CD28 ^null^ T cells accumulate as a result of increasing age ^71– 73^. The most conspicuous accumulations of CD28 ^null^ T cells occur in the CD8 compartment. These may be found in both young and older people and often constitute 20 to 80% of CD8 T cells. CD28 ^null^ CD4 T-cell frequencies are generally one if not two orders of magnitude lower than those of CD28 ^null^ CD8 T cells. Interestingly, their frequencies rarely exceed 1%, even in very old individuals, unless CMV infection is present. Our recent work indeed suggests that CMV infection is a major ‘trigger’ for the expansion of CD28 ^null^ CD4 T cells, increasing frequencies more than 10-fold on average. (In CD8 T cells, the effect was a twofold increase.) However, the mechanisms by which this happens have remained unclear. Our work also shows that chronological age has a very small effect on CD28 ^null^ CD4 T-cell expansions and only in CMV ^+^ individuals. Likewise, the effect of chronological age on the accumulation of CD28 ^null^ CD8 T cells was found to be quite small. Expansions of these cells have long been associated with CMV infection, but the strength of the association between large numbers of these cells and CMV infection was grossly underestimated. Only in the presence of CMV infection do individuals exhibit cell numbers typically associated with clinical disease in the literature (usually several percent) ^74^. A recent publication reported that treatment with ganciclovir in CMV ^+^ patients with anti-neutrophil cytoplasmic antibody (ANCA)-associated vasculitis reduced the number of CD28 ^null^ CD4 T cells ^75^. This finding seems to support both the ideas that these cells are driven by CMV infection and that their effects may be reversible when the offending antigen is removed ^75^.

According to our results and those of others, many CD28 ^null^ CD4 T cells are CMV-specific; however, it remains unclear whether other antigens are also recognised; no published report has ever convincingly shown the involvement of non-CMV antigens in triggering or driving CD28 ^null^ CD4 T-cell expansions.

Independently of any association with CMV, increased proportions of CD28 ^null^ CD4 T cells have been implicated in a wide range of autoimmune diseases over more than two decades but also in atherosclerosis and coronary heart disease. These different ‘strands’ of research appear to have existed independently of each other ^35^. We recently reviewed the literature on these topics and found compelling evidence that CMV is in all probability a major driver of vascular complications in RA and other chronic inflammatory conditions ^35, 76^. However, in extension of that work, we now explore the role of CD28 ^null^ CD4 T cells in autoimmune and chronic inflammatory diseases more generally. Our review brings together studies from different areas of autoimmunity all of which suggest a role for CD28 ^null^ CD4 T cells in causing or exacerbating disease ^77^. The presence of large numbers of CD28 ^null^ CD4 T cells in such conditions suggests a pathogenic role of this subset and indirectly one of CMV infection. Please note that most authors have reported increased proportions of these cells in various clinical situations rather than increased cell counts per unit of volume of blood (‘absolute counts’). Since lymphocytes may be redistributed between tissue and blood in different ways depending on a range of factors, absolute numbers per unit of blood are not necessarily more useful. In a recent publication, we were able to show that CMV-specific CD4 T cells drastically increase at older ages in both ‘absolute’ and relative terms; however, it also became clear that potentially relevant changes in subset proportions may be missed if the size of individual subsets is reported in ‘absolute’ counts ^78^.

## Rheumatoid arthritis

RA is a systemic autoimmune disease and the condition in which CD28 ^null^ CD4 T cells were first described and extensively studied ^21, 48, 49, 79^. A positive correlation between the presence of CD28 ^null^ CD4 T cells and the extent of extra-articular manifestations suggested that these cells mediate some of the systemic effects of RA ^48, 80^. It is unclear in which compartment these cells are originally generated. However, up-regulation of the chemokine receptor CX3CR1 on CD28 ^null^ CD4 T cells might allow them to reach the synovial fluid thanks to expression of the CX3CR1 ligand, fractalkine (CX3CL1), on synoviocytes ^81^. Remarkably, they are rarely found in the synovial membrane itself ^82^. Fractalkine and its receptor were previously proposed as promising candidates for anti-inflammatory interventions in RA but also cardiovascular disease and other chronic inflammatory conditions ^83^. In agreement with their role in systemic manifestations of RA, elevated levels of CD28 ^null^ CD4 T cells were found, for example, in inflammatory pulmonary infiltrates where they express increased levels of the α4 integrin (alpha4/beta1, CD11b) and CD11a ^20^. Expression of these markers likewise is likely to facilitate tissue infiltration. The RA-associated HLA-DRB1*04 allele group was previously linked to an increase in CD28 ^null^ CD4 T cells in both patients with RA and healthy controls ^23^. This might indicate that the presence of HLA-DRB1*04 alleles facilitates RA but also an increase in CD28 ^null^ CD4 T cells. Carrying HLA-DRB1*04 in that sense represents a predisposition to autoimmunity; as such, autoimmunity could be mediated at least in part by CD28 ^null^ CD4 T cells recognising a CMV epitope in this MHC context; however, to our knowledge, no CMV peptide presented by HLA-DRB1*04 has been reported to date. Our own work suggests that CMV infection is indeed the trigger for the expansions of these T cells.

The antigen specificity of CD28 ^null^ CD4 T cells is an area of great interest, but no antigens apart from CMV have been identified. Reactivity to CMV antigens was demonstrated in several studies, both in patients with RA and in healthy controls ^20, 82, 84^. Interestingly, both CMV DNA- and CMV-specific antibodies were reported in the synovial fluid of patients with RA ^85^.

**Figure 1.**
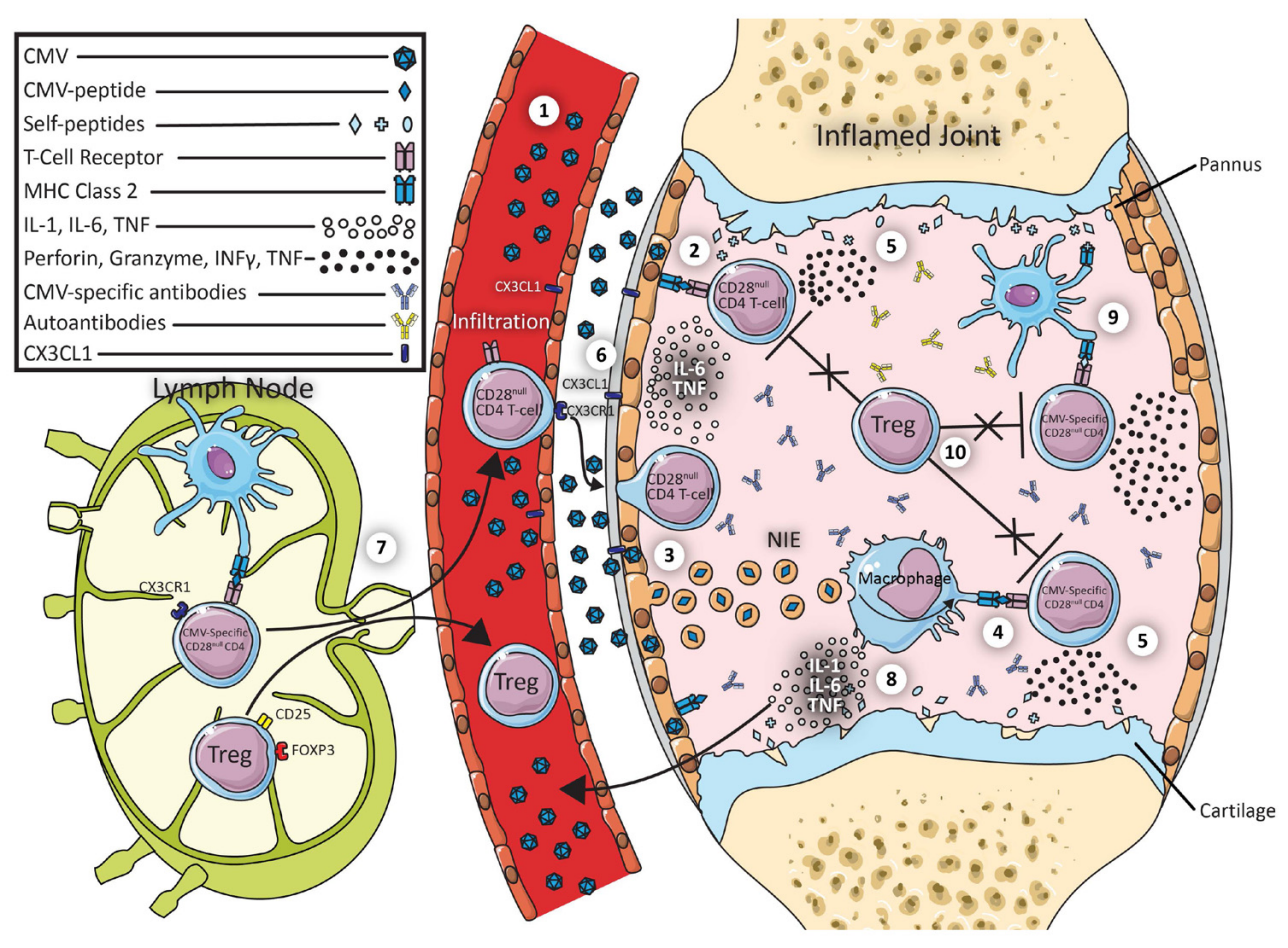
Potential link between CD28null T-cells, CMV, and Autoimmunity. **(1)** Endothelial cells (ECs) and synoviocytes can be infected by cytomegalovirus (CMV). **(2)** Synoviocytes may directly present CMV to CD28 ^null^ CD4 T cells in the context of class II major histocompatibility complex (MHC). **(3)** Synoviocytes may alternatively release non-infectious exosomes (NIEs) containing the CMV antigens (as shown for ECs). Antigen-presenting cells (APCs) will take up the NIEs and process the antigens to be presented on class II MHC. **(4)** APCs present CMV antigens to CMV-specific CD28 ^null^ CD4 T cells. **(5)** Activated CD28 ^null^ CD4 T cells produce interferon-gamma (IFN-γ) and tumour necrosis factor (TNF). **(6)** This in turn will up-regulate the expression of CX3CL1 on synoviocytes and ECs. **(7)** CX3CR1-expressing CD28 ^null^ CD4 T cells (and possibly regulatory T cells [Tregs]) travel from the lymph node to the inflamed joint (via the thoracic duct). **(8)** Interleukin-1 (IL-1) and TNF produced by activated macrophages cause tissue erosion and cartilage destruction. **(9)** Some CMV-specific CD28 ^null^ CD4 T cells may cross-react with self-antigens. **(10)** Activated CD28 ^null^ CD4 T cells evade suppression by Tregs and hence maintain an inflammatory state in the rheumatoid arthritis synovium.

This suggests that CMV might have a direct role in CD28 ^null^ CD4 T cell–mediated tissue damage in RA. Based on a number of publications, we have devised a hypothetical model how this might happen ( Figure 1).

We hypothesise that CMV-infected synovial fibroblasts directly present CMV antigens to infiltrating T cells in a class II MHC context. It has been shown that synovial fibroblasts can activate CD28 ^null^ CD4 T cells. However, the exact antigenic determinants remain unclear ^50^. Synovial fibroblasts might also present antigens indirectly via the release of non-infectious exosomes (NIEs) containing CMV antigens, as such a mechanism has been shown in endothelial cells (ECs). CMV-infected ECs release NIEs containing many CMV proteins. Following uptake of these NIEs by APCs, the CMV antigens are presented by MHC II on APCs to tissue-infiltrating CMV-specific CD4 ^+^ T cells ^86, 87^. Recognition of these antigens by the CD4 T cells would result in their activation and production of IFN-γ and TNF, both of which may up-regulate fractalkine expression on ECs and synoviocytes. This in turn would be likely to attract more CX3CR1-expressing CD28 ^null^ CD4 T cells and Tregs to the inflamed synovium. In addition, CMV-specific CD28 ^null^ CD4 T cells in the synovium might respond to autoantigens (as a result of cross-reactivity) and produce cytotoxic molecules like perforins and granzymes. CD28 ^null^ CD4 T cells might also activate macrophages to release IL-1 or TNF or both, which could stimulate synovial fibroblasts to cause cartilage erosion by promoting connective tissue growth factor ^88^. This is only a working model and, though speculative, is supported by the cited observations.

Apart from the adaptive immune response, macrophages and synovial fibroblasts might produce chemokines like IL-6, granulocyte-macrophage colony-stimulating factor (GM-CSF), and TNF in response to the presence of Toll-like receptor (TLR) ligands such as proteoglycans and, for example, bacterial DNA in the rheumatoid synovium ^89^. TLR-activated dendritic cells (DCs) migrate to the local lymph node to differentiate the primed autoreactive T cells towards different subtypes such as TH1 and TH2 (depending on the released cytokines) ^90^. A role of cytokines produced by macrophages and fibroblasts in autoimmune disease, in particular the role of TNF, has been well established. This is witnessed by the clinical success of anti-TNF therapies ^91^. Our model was conceived to illustrate one possible way how CMV infection might worsen the clinical course of RA via CD28 ^null^ CD4 T cells. We are aware that there may be other ways to explain the effect of CMV in autoimmune disease and a discussion/exploration of all these possibilities is urgently needed.

## Multiple sclerosis

MS is a debilitating autoimmune disease characterised by demyelination and axonal loss ^92^. Myelin-specific T cells have been shown to be associated with the neuronal damage in patients with MS. In addition, genetic predisposition and other environmental factors have been identified as contributors. There is a strong association of MS with the HLA-DR15 and HLA DQ6 alleles. Among the environmental triggers, vitamin D deficiency and Epstein–Barr virus have long been associated with the disease ^93^. Both CD4 ^+^ and CD8 ^+^ T cells have been proposed to play a role in disease progression.

Cytotoxic CD28 ^null^ CD4 T cells are indeed found in increased proportions in patients with MS. They display up-regulated cell adhesion molecules, which probably allow them to infiltrate the brain and destroy oligodendrocytes ^34, 41, 94, 95^. Thewissen *et al*. reported that CD28 ^null^ CD4 T-cell reactivity against myelin basic protein (MBP) in patients with MS was limited ^20^. However, in another study, cross-reactivity between a myelin peptide (MOG 35-55) and a CMV antigen UL-86 (981-1003) was observed in both rats and non-human primates ^42^.

In a recent publication, CMV seropositivity of patients with MS was associated with higher percentages of CD28 ^null^ CD4 T cells ^34^. Although no direct evidence of an association of CD28 ^null^ CD4 T cells with MS severity was found in humans, the authors observed expansion of CD28 ^null^ CD4 T cells in response to CMV infection in a mouse CMV (MCMV) model. There was a direct correlation between the frequencies of CD28 ^null^ CD4 T cells and disease severity in a mouse model of MS, referred to as experimental autoimmune encephalitis (EAE) ^34^. Infection of EAE mice with MCMV further increased the frequencies of CD28 ^null^ CD4 T cells. In these mouse models, CD28 ^null^ CD4 T cells could also be induced by repeated stimulation of T cells with MBP or tetanus toxoid *in vitro*, indicating that the CD28 ^null^ phenotype of CD4 T cells is not strictly antigen-dependent in these animals. Antigens other than CMV inducing this phenotype in humans, however, have not yet been identified. It is of note that CMV-specific CD8 T cells were previously reported in MS lesions ^96^.

## Systemic lupus erythematosus

Systemic lupus erythematosus (SLE) affects multiple organs and is associated with increased mortality and morbidity. Most patients with SLE have sera positive for autoantibodies against La protein, which is a small ribonucleoprotein (RNP) associated with RNA polymerase. Interestingly, many viruses use La protein for their replication. A recent review on CMV infection in childhood-onset SLE describes CMV to either be the trigger of the disease or be responsible for a flare. This hypothesis is based on the association of La autoantibodies and latent CMV infection in many patients ^97^. Another study identified antibodies against the 47-kD La protein (SS-B) that recognised epitopes exhibiting sequence similarity with many herpes viruses, including CMV ^98^. Recently, the role of T cells in SLE has been recognised; T cells in SLE were shown to have a lower threshold for activation. Different mechanisms by which these T cells lower their activation threshold were discussed in a recent review by Mak and Kow ^99^. T cells in patients with SLE were shown to have decreased levels of CD3 ζ-chain (zeta chain) expression, measured by anti-CD3-ζ immunoblots, as compared with healthy controls. These so-called ‘lupus T cells’ were predominantly CD8 ^+^ and CD16 ^+^^100^. Decreased levels of CD3 ζ-chain expression leads to a rewiring of the downstream ZaP-70 signalling pathway. This causes an increased calcium flux and greater activation of these T cells ^99^. Further work is required to characterise these T cells with regard to other co-stimulatory molecules, especially CD28, in the context of CMV infection. Also, it would be interesting to assess the potential cross-reactivity of CMV-reactive T cells with La protein.

## Graves’ disease

GD is an autoimmune disease that affects the thyroid glands. Anti-thyrotropin-stimulating receptor antibodies were found to cause goitre and hyperthyroidism in these patients. A recent study found increased levels of CD28 ^null^ CD4 T cells in GD patients compared with healthy controls. The same study found that the numbers of CD28 ^null^ CD4 T cells correlated with the severity of the goitre and the ophthalmopathy. In patients with goitre-like symptoms, 15% of the total lymphocytes were CD28 ^null^ CD4 T cells compared with only 6% in patients without goitre-like symptoms ^22^. Giving further support to a role of these T cells, the percentages of IFN-γ-producing CD28 ^null^ CD4 T cells correlated with those of anti-thyrotropin-stimulating receptor autoantibodies (r = 0.4581, *P* = 0.002).

IFN-γ-producing CD28 ^null^ CD4 T cells were found not only in the circulation but also, in high numbers, in affected tissues such as the muscles in the eye and the thyroid ^59, 101^. High levels of circulating IFN-γ were observed in patients with GD and this might drive the destruction of thyroid tissue ^22, 60^.

The role of virus infections in GD has been addressed in previous studies. For example, antibodies against the capsid antigens of Epstein–Barr virus were found at higher titers ^102^. However, the sera of these patients were not examined for anti-CMV antibodies. Since CMV is the driver of the CD28 ^null^ CD4 T-cell population, a possible role for CMV in GD should be investigated.

## Therapies targeting CD28 ^null^ CD4 T cells

The consistent presence of potentially highly pathogenic CD28 ^null^ CD4 T cells across different autoimmune diseases potentially makes them an interesting target for immunotherapy, and some immunotherapies that are currently in clinical use were shown to have effects on this subset, such as reducing its frequency or reversing its phenotype by causing re-expression of CD28. For example, anti-TNF-α therapy, which is widely used in the treatment of RA, was shown to limit the expansion and cytotoxic effects of CD28 ^null^ CD4 T cells ^32^. Discontinuation of anti-TNF conversely may increase the risk of disease flares ^103^. Methotrexate leads to a reduction of CD28 ^null^ CD4 T-cell levels, whereas infliximab induces the re-expression of CD28 ^51^. Others have shown re-expression of CD28 by IL-12 stimulation ^38^. However, it remains unclear at this time what effect, for example, re-expression of CD28 might have.

CD28 ^null^ CD4 T cells are generally considered senescent cells as they have lost the ability to proliferate. Senescent cells usually have shortened telomeres, which activate the DNA damage repair (DDR) pathway. This pathway allows cells to be eliminated before they can pose a threat to the organism. (The mechanisms of senescence and exhaustion of T cells were reviewed in great detail in a recent review ^104^.) However, it appears that therapies aiming to reverse the senescence of CD28 ^null^ CD4 T cells could be helpful for improving vaccination results. On the other hand, this might carry the risk of generating malignant cells. In addition, we believe it cannot be fully excluded that re-activating senescent CD28 ^null^ CD4 T cells could worsen some of the conditions associated with their expansion. This could be the result of allowing even further proliferation of the then ‘reverted’ cells which now would be expressing CD28. (This is implying that in some situations senescent CD28 ^null^ T cells could do less harm than their CD28 ^+^ counterparts.)

Therefore, therapies removing CD28 ^null^ CD4 T cells might be a safer option and more beneficial. In theory, this could be achieved by targeting specific molecules on the CD28 ^null^ CD4 T subset or by inducing apoptosis specifically of this subset. For example, apoptosis of CD28 ^null^ CD4 T cells has been achieved by using the lipid-lowering rosuvastatin in patients with coronary heart disease ^105^. To reduce the tissue-infiltrating capacity of CD28 ^null^ CD4 T cells, Broux *et al*. have suggested blocking CX3CR1 on these cells ^41^. It will be important for the success of any of these therapies to characterise CD28 ^null^ CD4 T cells in more detail and to identify both their antigenic specificity and molecular markers. Additionally, antiviral therapies such as ganciclovir could potentially help to reduce the expansion of the CD28 ^null^ CD4 T cells and hence possibly reduce the severity of many of the autoimmune diseases. However, the way in which such a strategy might be used clinically is, in reality, far from clear.

## Conclusion and future perspective

CD28 ^null^ CD4 T cells are an unusual population of chronically stimulated cells. In humans, they appear to accumulate in the presence of CMV infection. They are cytotoxic, produce large quantities of cytokines (a secretory phenotype associated with senescence) and display increased levels of adhesion markers, which suggests that they have access to diverse tissues. Their mere presence in autoimmune disease does not prove that they are actually involved in it or even cause it. However, reports indicating a correlation of their frequencies with severity suggest direct involvement in causing pathology. Studies in which these cells are specifically targeted might show to what extent they are involved in disease progression. Understanding the different antigenic specificities of these cells is a major challenge but will be helpful for developing specific immunotherapies. CMV is the clear trigger for the accumulation of these cells. However, we still do not know exactly which antigens (apart from CMV) these cells recognise. A better understanding of the antigen specificity of CD28 ^null^ CD4 T cells would be a big step forward.
